# Fluorescently Labelled Silica Coated Gold Nanoparticles as Fiducial Markers for Correlative Light and Electron Microscopy

**DOI:** 10.1038/s41598-018-31836-1

**Published:** 2018-09-11

**Authors:** Jantina Fokkema, Job Fermie, Nalan Liv, Dave J. van den Heuvel, Tom O. M. Konings, Gerhard A. Blab, Andries Meijerink, Judith Klumperman, Hans C. Gerritsen

**Affiliations:** 1 Soft Condensed Matter and Biophysics, Debye Institute for Nanomaterials Science, Utrecht University, Utrecht, The Netherlands; 2 Section Cell Biology, Center for Molecular Medicine, University Medical Center Utrecht, Utrecht University, Utrecht, The Netherlands; 3 Condensed Matter and Interfaces, Debye Institute for Nanomaterials Science, Utrecht University, Utrecht, The Netherlands

## Abstract

In this work, gold nanoparticles coated with a fluorescently labelled (rhodamine B) silica shell are presented as fiducial markers for correlative light and electron microscopy (CLEM). The synthesis of the particles is optimized to obtain homogeneous, spherical core-shell particles of arbitrary size. Next, particles labelled with different fluorophore densities are characterized to determine under which conditions bright and (photo)stable particles can be obtained. 2 and 3D CLEM examples are presented where optimized particles are used for correlation. In the 2D example, fiducials are added to a cryosection of cells whereas in the 3D example cells are imaged after endocytosis of the fiducials. Both examples demonstrate that the particles are clearly visible in both modalities and can be used for correlation. Additionally, the recognizable core-shell structure of the fiducials proves to be very powerful in electron microscopy: it makes it possible to irrefutably identify the particles and makes it easy to accurately determine the center of the fiducials.

## Introduction

The field of correlative light and electron microscopy, or CLEM, has expanded rapidly during the last decade. Especially in biology it turns out to be very useful to combine these two techniques. Light microscopy or fluorescence microscopy (FM) is used to visualize, localize and track specific fluorescent molecules in cells over large areas with high sensitivity, while electron microscopy (EM) provides high resolution ultrastructural information of cells and materials^1,2^. This opens up the possibility to visualize rare transient events or specific cells within complex tissues^3,4^.

For the best results in CLEM experiments, data from the different modalities should be registered with the highest possible precision. This is complicated by the vastly different fields of view of FM and EM, as well as the different contrast mechanisms of these techniques. FM requires bright and stable fluorophores, while EM relies on differences in electron density for contrast, and frequently requires heavy metal staining to visualize biological structures. Since fluorescent probes (i.e. molecules or proteins) are typically not electron dense, fluorescent labels can generally not be used for correlation.

Particles visible in both modalities (fiducial markers) can be used to overcome this problem. The viability of this approach has been demonstrated in literature by using fluorescent latex beads^5–7^ or quantum dots^8–10^. However, a shared problem of these candidate particles is their relatively low EM contrast, making visualization and localization in heavily EM stained samples difficult or even impossible. An alternative approach to register data between modalities is via a double labelling procedure. Here, proteins of interest are labelled with a fluorescent probe, followed by labelling with antibodies or protein A conjugated with colloidal gold^11^. A disadvantage of this approach is that correlation is indirect and based on the assumption that both labels fully colocalise. Despite great successes achieved by this approach, Miles *et al*.^12^ recently demonstrated that this assumption not always holds true, thereby stressing the importance of finding a more direct way for registering FM and EM data.

**Figure 1:**
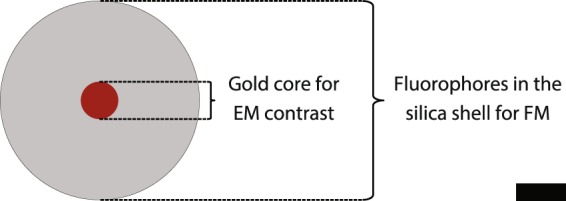
A schematic representation of the fiducial markers. The scale bar indicates 20 nm.

In this work, nanocomposite core-shell particles based on a gold core and a fluorescently labelled silica shell (Fig. 1) are deployed as fiducial markers. The gold core provides contrast for EM and fluorophores covalently incorporated in the silica shell for FM. Rhodamine B is chosen as fluorophore because it was demonstrated by Karreman *et al*.^13^ that rhodamine like fluorophores behave well under the dry and vacuum conditions encountered in EM. Using a red emitting fluorophore is also advantageous because excitation at longer wavelengths results in reduced autofluorescence^14^. To obtain the fiducials, first, an optimized synthesis of the nanocomposite particles to obtain spherical and highly monodisperse particles of arbitrary size is presented. Next, a thorough study is performed to optimize the fluorophore labelling density within the silica shell to obtain bright and (photo)stable particles. Finally, the particles are tested as fiducials in a 2D and a 3D CLEM experiment.

## Results

### Optimization of fluorophore density

**Figure 2:**
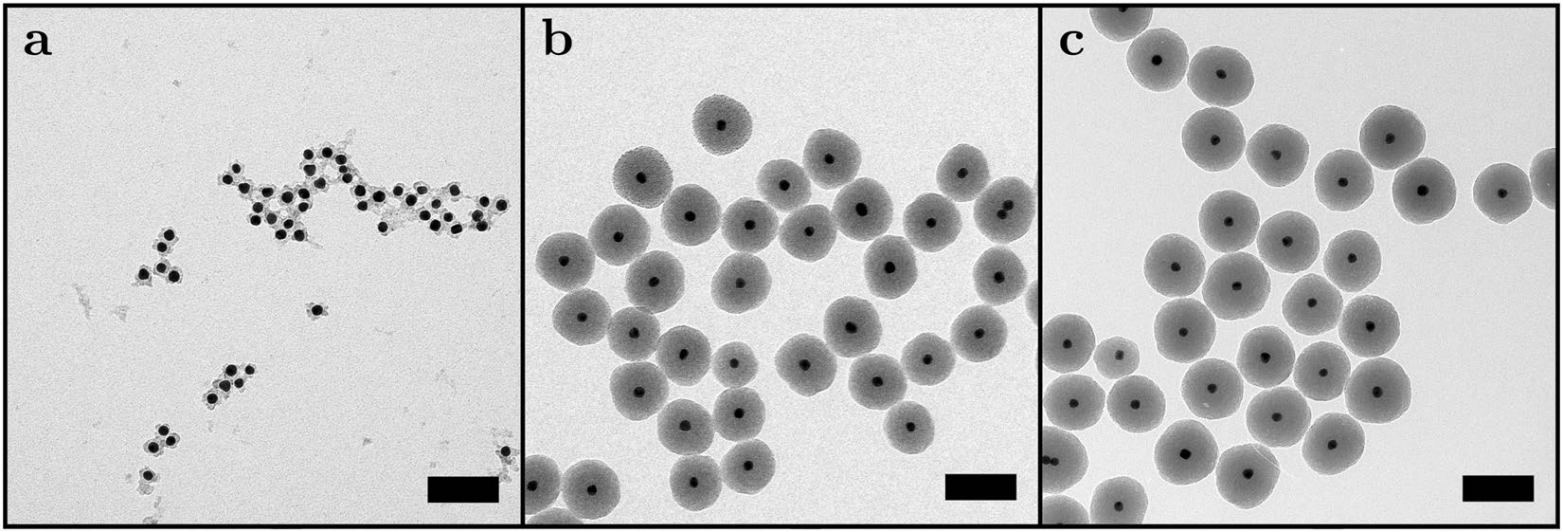
Representative TEM images at different stages of the silica coating procedure; (**a**) after growth of the first stabilization layer; (**b**) after growth of the rhodamine B labelled silica layer; (**c**) after growth of the second stabilization layer. The scale bars indicate 100 nm.

A series of ~90 nm diameter particles with relative dye concentrations of 0 to 30 were synthesized, see Table 1 and SI-1. Transmission electron microscopy (TEM) measurements were carried out to determine particle sizes at different stages of the synthesis. Representative images of particles are shown in Fig. 2. The images illustrate that the particles are successfully coated with a very thin silica layer after the first growth step (a). The silica layer becomes thicker and more homogeneous after growth of the rhodamine B labelled silica layer (b) and the second stabilization layer (c). It is important to note that silica grows selectively onto the existing particles, i.e. no secondary nucleation takes place. Average particle diameters and standard deviations at the different stages of the synthesis were determined from TEM images. Average particle sizes are almost identical for all samples at different stages of the reaction, justifying the assumption that the volume of rhodamine B labelled silica per particle is similar for all samples. By ensuring that the number of particles is the same in all reactions, it is ensured that we are truly studying fluorophore labelling density effects.

**Table 1:** Particle sizes determined from TEM after growth of the rhodamine B labelled silica layer and growth of the second stabilization layer and corresponding fluorophore labelling efficiencies and average separations between fluorophores.

[Dye]	Total particle diameter				Labelling efficiency (%)	Average separation (nm)	Fluorophores per particle (a.u.)
	RITC layer		Stab. layer				
	d (nm)	*σ* (nm)	d (nm)	*σ* (nm)			
30	79	4.5	89	4.9	3.7	7.7	558 ± 24
25	86	5.4	88	4.8	4.1	7.9	662 ± 26
20	83	5.0	88	5.4	4.5	8.2	530 ± 23
15	82	4.3	88	4.8	4.3	9.2	358 ± 19
12.5	82	4.3	87	5.0	3.8	10.2	265 ± 16
10	82	4.7	89	4.5	3.2	11.7	175 ± 13
7.5	83	4.8	89	4.7	3.5	12.4	151 ± 12
5	81	4.7	88	5.1	2.9	15.2	77 ± 9
2.5	83	5.6	89	5.3	2.7	19.6	39 ± 6
1	82	4.8	89	4.5	2.2	28.5	12 ± 4
0	76	4.8	89	5.4	N/A	N/A	N/A

All samples were based on 15 nm (*σ* = 1.4 nm) diameter gold cores coated with a thin silica shell to obtain a total diameter of 25 nm (*σ* = 2.7 nm). In this table, d is the average particle diameter and *σ* is the standard deviation of this average value, both were determined by measuring diameters of 100 particles.

To calculate the average fluorophore separations, fluorophore labelling efficiencies were determined. This was performed via absorption measurements after dissolution of the silica shell of the particles as described by Imhof *et al*.^15^. Next, the average separation between fluorophores was calculated as the cube root of volume available per fluorophore. This value increases from 7.7 nm for the highest to 28.5 nm for the lowest labelling density (see Table 1). The estimated average number and corresponding error $(\sqrt{N})$ of fluorophores per particle ranges from 12 for the lowest to 558 for the highest labelling density and is also included in this Table.

#### Spectral measurements and radiative decay curves

**Figure 3:**
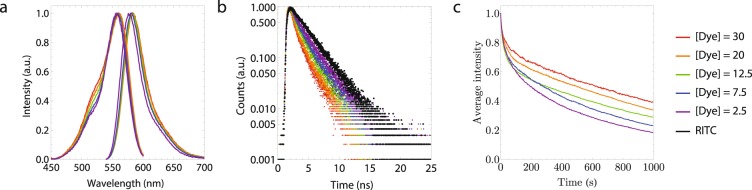
(**a**) Emission spectra recorded after excitation at 520 nm (right) and excitation spectra recorded at 610 nm (left). (**b**) Radiative decay curves after pulsed excitation at 509 nm and detection at maximum emission. (**c**) Normalized bleaching curves measured at an excitation intensity of 6.0 W cm^−2^.

To study fluorophore density effects, excitation and emission spectra and radiative decay curves were recorded (see Fig. 3a,b). Both spectra exhibit a small blue shift with increasing fluorophore density that is accompanied by an increase in the height of the shoulder around 520 nm in the excitation spectrum. Radiative (or fluorescence) decay curves were measured using the time-correlated single-photon counting technique after excitation with a pulsed laser^16^. These curves reveal faster decays with increasing labelling density which is indicative for fluorescence quenching. The radiative decay of particles labelled with the lowest dye labelling density, [Dye] = 2.5, is already slightly faster than the decay of the fluorophore, rhodamine B isothiocyanate (RITC), in ethanol. This can be attributed to a change in the local medium of the fluorophores, silica versus ethanol, and the APTES-dye coupling.

Similar observations at increasing fluorophore densities in solid matrices were made by Genovese *et al*.^17^ (rhodamine B in pluronic silica) and Imhof *et al*.^15^ (fluorescein in Stöber silica) and for fluorophores on antibodies by Szabó *et al*.^18^. An explanation for these observations can be found by taking into account concentration effects including self quenching. Increasing the fluorophore density results in the formation of an increased number of dimers^19,20^ or other species acting as quenching centers. Additionally, resonance energy transfer (homo-FRET) between fluorophores becomes more efficient because the average separation between fluorophores shortens^16^. This combination of energy transfer and quenching successfully explains the effects of fluorophore concentration on the fluorescence quantum yield of fluorophores in solution^21,22^ and can also be applied to the work presented here. When increasing the fluorophore density in the particles, energy transfer between fluorophores becomes possible and the number of quenching centers within the particles increases. This energy transfer allows the excited state to migrate within the particle which makes it possible for the excited state to migrate from an unquenched fluorophore to a nearby quenching center, thereby contributing to quenching of fluorescence.

#### Single particle intensity and (photo)chemical stability

**Figure 4:**
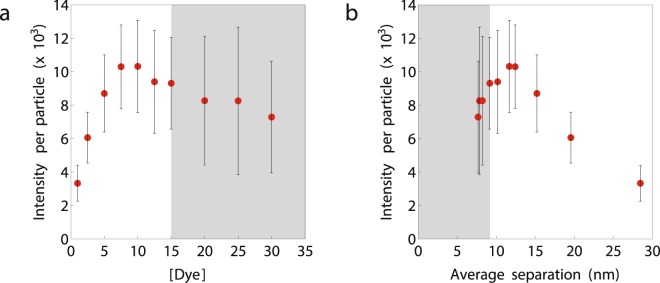
Average single particle intensity plotted as a function of the relative dye concentration (**a**) and as a function of the average fluorophore separation (**b**). The standard deviation in this plot corresponds to the standard deviation of the normal distribution that was fitted to the data to obtain the mean intensity. The area in gray corresponds to non-stable particles.

Widefield fluoresence microscopy measurements were performed to determine the single particle intensity and photostability of the particles. In Fig. 4a the average single particle intensity is plotted as a function of the relative dye concentration. It can be seen that initially the single particle intensity increases with increasing dye concentration. After this first increase, the single particle intensity remains more constant and eventually a small drop in intensity is observed. This optimum can be explained by the counterbalance between increasing the number of fluorophores versus the increase in self quenching which becomes more evident when the single particle intensity is plotted as a function of the average fluorophore separation, see Fig. 4b. At large separations (>15 nm) the single particle intensity increases when the number of fluorophores per particle increases (i.e. shortening of the average separation) because the fluorophores do not sense each other. Next, a more constant regime between 10–15 nm with an optimum around 12 nm is observed. This constant regime can be explained by the counterbalance between increasing the number of fluorophores versus the increase in self quenching. Finally, when average separations drop below 10 nm self quenching becomes dominant which results in the aforementioned drop of intensity.

The photostability of the particles was studied by measuring their photobleaching behavior. From the photobleaching curves in Fig. 3c it becomes clear that, generally speaking, the intensity loss reduces when the fluorophore density increases. This can be explained by the shortening of the radiative decay time at higher fluorophore densities. The fluorophores spend less time in the excited state, thereby decreasing the bleach rate. In addition, photobleaching reduces the effective dye concentrations which decreases the effect of concentration quenching thereby counterbalancing bleaching.

Another observations made throughout the experiments was that particles with a relative dye concentration above 15 tend to cluster over time (gray area, Fig. 4). This clustering can result from a reduction of the negative zeta potential caused by the incorporation of positively charged fluorophores and amine groups. For CLEM applications non-clustered particles are preferred, therefore these high concentrations should be avoided.

Based on the results included in this section, a relative fluorophore concentration of 10 (separation 11.7 nm) was chosen as the optimum labelling density. The average single particle intensity is maximum around this concentration. In terms of bleaching, it might be desirable to go for a higher labelling density. However, because of particle stability, a relative fluorophore concentration well below 15 (separation 9.2 nm) is desired.

### 2D CLEM experiment: Widefield and TEM imaging of fiducials on thin cryosections

**Figure 5:**
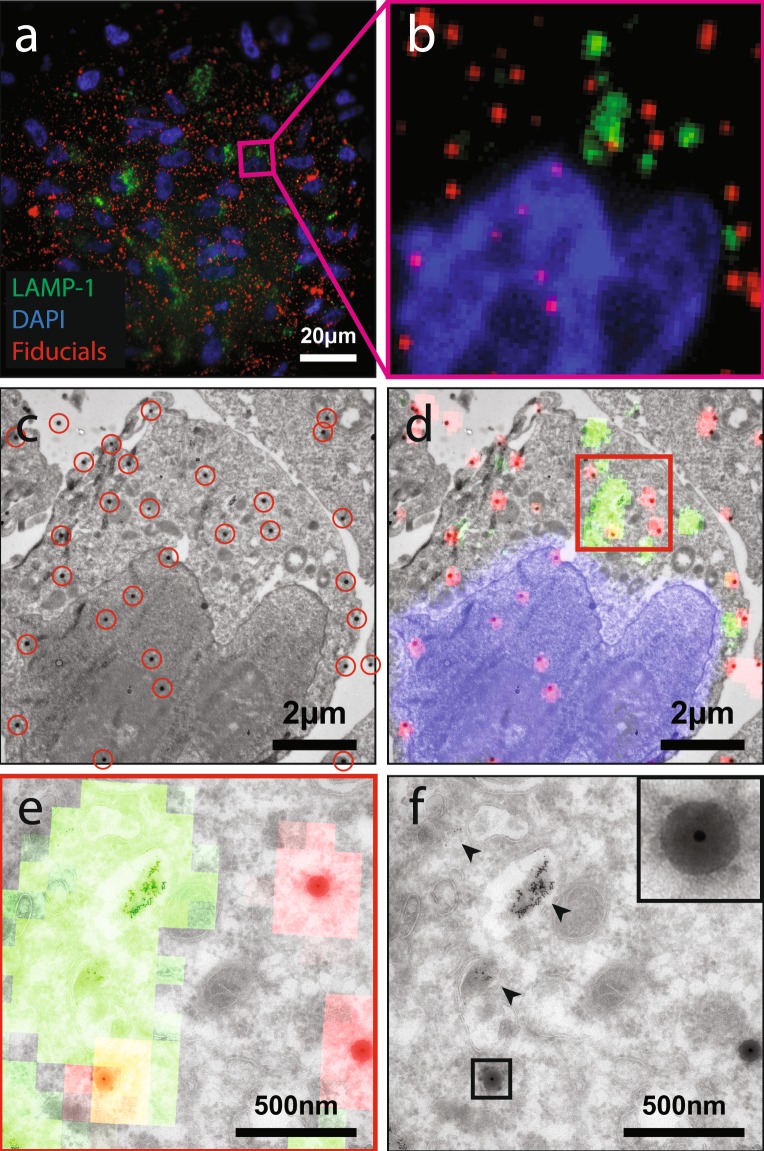
2D CLEM experiment of 81.9 nm diameter fiducials on top of a 70 nm thick cryosection of HT1080 cells stably expressing LAMP-1-GFP. (**a**) Widefield fluorescence microscopy image. (**b**) Region selected for TEM imaging. (**c**) TEM image of the selected region (fiducials encircled in red). (**d**) Overlay of FM and EM data based on the positions of the particles. In (**e**) and (**f**) higher magnification CLEM and TEM images of the in (**d**) highlighted region are included. The GFP fluorescence perfectly overlaps with the position of late endosomes and lysosomes, which were identified in TEM by their ultrastructural characteristics and the presence of endocytosed BSA-Au^5^ (arrowheads). In the inset of (**f**) one of the fiducials is enlarged, highlighting the core-shell structure of the particles.

The nanocomposite particles with the optimum dye concentration were first tested as fiducials in a 2D CLEM experiment. This experiment was performed by the addition of the particles to a cryosection of cells on a TEM grid in a correlative workflow. Results of this experiment are included in Fig. 5 and demonstrate that the fiducials are clearly visible in both modalities. The FM/EM overlay included in (d) and (e) is purely based on the positions of the fiducials and is used to correlate LAMP-1-GFP fluorescence to the ultrastructure of late endosomes and lysosomes. The inset in Fig. 5f clearly shows the core-shell structure of the fiducials. This core-shell structure was also apparent at the lower magnification TEM image in Fig. 5 and proved to be very useful to identify the fiducials; additional images are included in the supplementary information (SI-3). The core-shell also proved to be very useful to accurately determine the center of the fiducials and having a distinguishable well defined structure opens up possibilities for automatic registration of the particles. It should be noted that the EM magnification should be high enough to observe the core-shell structure of the fiducials. At too low magnifications, the core-shell structure is no longer visible which complicates discriminating fiducials from dirt and automatic registration of the fiducials.

### 3D CLEM experiment: Endycotised nanoparticles as fiducials for correlative confocal fluorescence and 3D electron microscopy

In biological samples, correlating regions of interest (ROIs) between fluorescence and electron imaging can prove challenging due to the heterogeneous content of the cell and limited resolution of fluorescence imaging. Several organelles can be located within the same fluorescent spot, causing the risk of misidentification. The use of fiducials improves the registration accuracy between FM and EM, and can aid in alleviating this issue, especially when imaging in 3D. Due to their unique gold core silica shell architecture, we hypothesized that the nanoparticles could function as well-defined fiducials to correlate fluorescence and 3D electron imaging data. Previous research on silica particles has demonstrated that, under the right conditions, cells readily take up silica particles through endocytosis without cytotoxic effects^23–26^, indicating that endocytosed particles could serve as a useful and functional fiducial.

**Figure 6:**
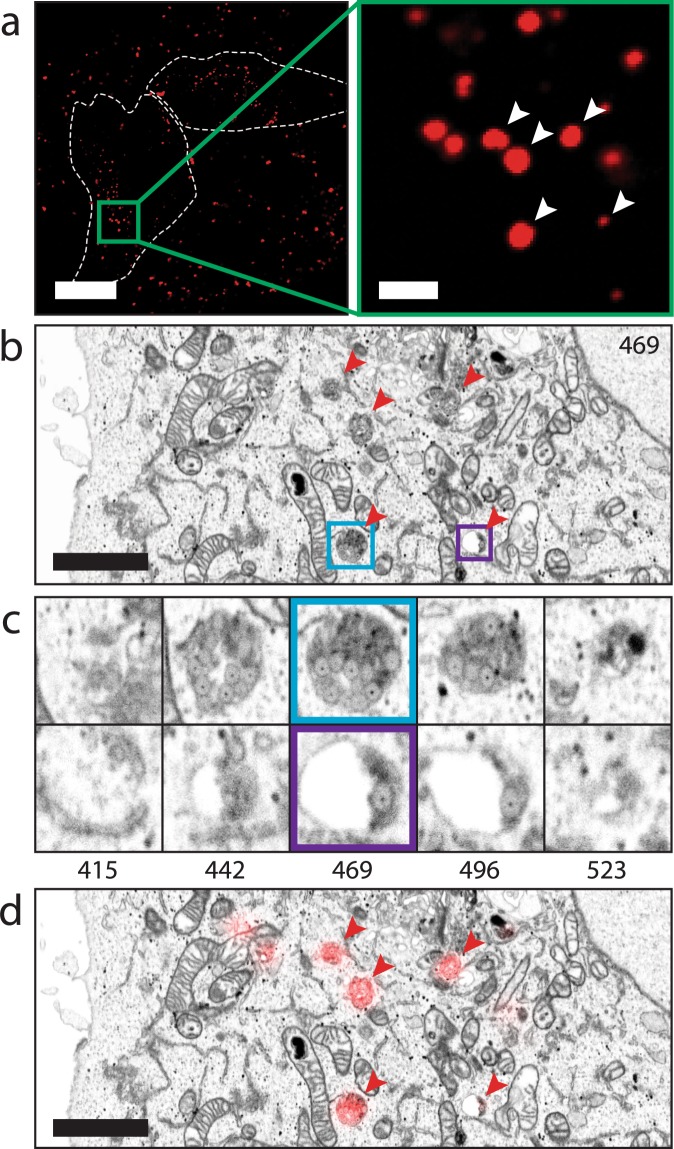
3D CLEM experiment of 99.2 nm diameter fiducials after cellular uptake in HeLa cells. (**a**) Confocal fluorescence microscopy slice of cells after fixation. White areas indicate coarse outlines of two cells. The enlarged region highlighted in green is chosen as ROI for FIB-SEM imaging. The white arrowheads indicate fiducials visible in the corresponding FIB-SEM slice shown in (**b**). (**b**) Virtual slice of the reconstructed FIB-SEM data, corresponding to the focal plane used for fluorescence imaging. Red arrowheads indicate compartments visualized in fluorescence microscopy, which contain various amounts of fiducials. The organelles encircled in blue and purple containing fiducials are enlarged in (**c**). (**c**) FIB-SEM slices through organelles of interest at different heights of both compartments, clearly showing the different amounts of fiducials present. The numbers below the figures correspond to the number of the FIB-SEM slices. (**d**) Overlay of FM and EM data based on the positions of the fiducials indicated by the arrowheads in (**a,b**). The scale bars in (**a**) correspond to 20 and 2 *μ*m, the scale bars in (**b,d**) correspond to 2 *μ*m.

To examine the viability of the nanoparticles as 3D fiducials, we incubated HeLa cells with the nanoparticles diluted in medium, allowing uptake of the particles into the cells. After three hours the samples were fixed and imaged using confocal fluorescence microscopy. Endocytosed nanoparticles were detected throughout the cells (Fig. 6a), indicating successful endocytosis. Following fluorescence imaging, we selected a region of a cell containing both large, bright spots and smaller, dimly fluorescent spots for FIB-SEM imaging (Fig. 6a, inset). Samples were postfixed, stained and embedded for imaging by focused ion beam scanning electron microscopy (FIB-SEM). In FIB-SEM, samples are imaged by scanning the surface of a ROI using the electron beam, after which a thin layer is ablated from the surface using the FIB. This cycle is repeated until the ROI has been imaged, allowing 3D reconstruction of a sample. FIB-SEM on biological samples requires relatively severe staining with heavy metals to obtain sufficient detail of cellular structures, which comes at the risk of obscuring fiducials, and exaggerating biological features that may be mistaken for fiducials. In our FIB-SEM data, we found that the combination of the electron-dense gold core and the electron-lucent silica shell made for easy, unequivocal identification of the compartments containing nanoparticles, even in heavily stained samples, allowing easy correlation of fluorescence and FIB-SEM data.

The nanoparticles were found in endocytic compartments (Fig. 6b), and could be resolved at an individual particle level. The unique structure of the particles proved helpful in identification, meaning that any fluorescent spot seen in the confocal data could be linked to corresponding particles detected using FIB-SEM (Fig. 5b). Interestingly, brightly fluorescent spots were correlated to compartments containing up to 40 particles (Fig. 6c, blue outline), whereas only 1 or 2 nanoparticles could be found in compartments corresponding to dimly fluorescent spots (Fig. 6c, purple outline), indicating a high level of sensitivity. Thanks to the high resolution and small sectioning distance employed by FIB-SEM, even single particles could be detected, and fitted to the fluorescent data. In the FM (Fig. 6a) and FIB-SEM data, particles, and clusters of particles were also observed outside the cells. Thorough testing, including Dynamic Light Scattering measurements, proved that initially non-clustered particles were presented to the cells. The observation of clusters outside the cells can be explained by taking into account that this data is recorded after 3 hours of uptake, after the addition of fixative and after overnight fixation. Within this time frame, particles still present in the medium can aggregate, in particular after the addition of fixative. This aggregation is not expected to affect the initial uptake of the particles but this could complicate automatic registration strategies.

Combined, our data shows that the nanoparticles are taken up by HeLa cells and are useable as 3D fiducials, owing to their bright fluorescence and ease of identification in FIB-SEM data.

## Conclusions and Outlook

15 nm gold particles coated with a fluorescently labelled silica shell (rhodamine B isothiocyanate (RITC)) were successfully synthesized. A relative dye concentration of 10, corresponding to an average fluorophore-fluorophore separation of 11.7 nm yielded optimum brightness and (photo)stability. Particles labelled with this optimum dye concentration were successfully used as fiducials in a 2D CLEM experiment to correlate widefield FM and TEM images by addition of the fiducials to a cryosection of cells on a TEM grid, demonstrating high registration accuracy in both FM and EM. After endocytosis of the fiducials by HeLa cells, the particles could also be used as well-defined fiducials to correlate confocal FM and FIB-SEM. In both experiments, the unique core-shell signature of the fiducials proved very useful to identify the fiducials and to accurately determine the center of the fiducials. This was especially evident in the FIB-SEM data, where a fiducial with only an electron-lucent shell or only a small electron-dense core would be at risk of being misidentified as a cellular structure.

In future research, automatic registration procedures will be explored were the distinct core-shell structure of the here presented particles can be used to detect the fiducials in EM. Furthermore, we plan to use the offset between EM and FM positions of the fiducials to correct for FM/EM sample distortions. This opens up the possibility to use the fiducials to test and quantify the accuracy of different data correlation methods. Such a method can for example include nonlinear effects such as sample deformation caused by shrinkage of the sample in EM. Furthermore, the unique architecture of the nanoparticles can aid in devising automated correlation strategies, based on accurate localization of the nanoparticles within complex biological specimens. Finally, we note that due to the silica shell the particles are non-toxic and compatible with live cell imaging experiments, opening up imaging strategies for live-cell correlative imaging.

## Materials and Methods

### Synthesis of the fiducial markers

The fiducial markers used in this study were synthesized via a multistep procedure, experimental details are included in the supplementary information (SI-1 and 2). Briefly, gold particles with a diameter around 15 nm were synthesized via the sodium citrate reduction^27,28^. After polyvinylpyrrolidone (PVP) functionalization^29^, the particles were transferred to ethanol and coated with a very thin non fluorescent silica layer using a seeded growth procedure based on the traditional Stöber method^30–32^. This layer stabilizes the particles and acts as a spacer between the gold and the fluorophores. Next, the particles were coated with a rhodamine B labelled silica layer. By coupling the fluorophores to (3-aminopropyl)triethoxysilane (APTES) molecules prior to the synthesis, fluorophores were covalently incorporated within the silica matrix^15,33^. Finally, to keep the particles stable, the particles were coated with a second thin silica layer. To optimize the fluorophore labelling density, particles labelled with different fluorophore densities were synthesized by varying the amount of APTES-fluorophore complex added during growth of the fluorescent silica layer. The particles were characterized to find the optimum labelling density in terms of particle brightness and (photo)chemical stability.

### Determination of the fluorophore incorporation efficiency

Immediately after synthesis of the particles, 3 mL of the reaction mixture was transferred to a 5 mL eppendorf tube. This solution was centrifuged 15 minutes at 15.000 rcf to separate the particles from the reaction mixture. The supernatant was collected and stored. The particles were redispersed in 3 mL absolute ethanol and centrifugation and redispersion in ethanol was repeated two more times. 1.5 mL particle solution and 1.5 mL of a 0.4 M sodium hydroxide solution in water were transferred to a clean 5 mL eppendorf tube. After homogenization, solutions were stored for 48 hours to ensure complete dissolution of the silica shell of the particles. Next, the solutions were centrifuged 30 minutes at 20.000 rcf to remove the non-dissolved gold cores from the solution. Supernatants, ranging from transparent and colorless for the blanco ([Dye] = 0] to transparant pink for high labelling densities ([Dye] = 30) were separated from the red to black pellets and stored. Absorption spectra of all solutions were recorded on a HP8953A spectrophotometer in 1 cm quartz cuvettes. If necessary, samples were diluted with a 1:1 (volume) mixture of ethanol and 0.4 M sodium hydroxide solution.

### Spectral and radiative decay measurements

Bulk excitation and emission spectra and radiative decay measurements of the particles suspended in ethanol were recorded in 1 cm quartz cuvettes using an Edinburg Instruments FLS920 fluorescence spectrometer. In all measurements, fluorescence was detected at an angle of 90° to the exciting beam. Furthermore, a 530 nm longpass filter was placed between the sample and the detector in all measurements to remove residual excitation light. To record excitation and emission spectra, a 450 W xenon lamp and a double excitation monochromator with a grating blazed at 500 nm was used for excitation. Spectra were recorded with a Hamamatsu H74220-60 photo sensor module with a grating blazed at 500 nm. For the radiative decay measurements a picosecond pulsed diode laser (EPL-515) emitting at 509.8 nm with a 50 ns pulse period and a 204.4 ps pulse width was used for excitation. Radiative decay curves were recorded with a Hamamatsu R928 PMT detector with a grating blazed at 500 nm.

### Single particle measurements

Measurements were carried out on a Nikon Eclipse Ti widefield microscope equipped with a 40 × 0.75 NA Nikon air objective. A Nikon TI-ND6-PFS perfect focus unit was used to retain sample focus during the measurements. A mercury arc lamp in conjunction with a 510–560 nm excitation filter, a 565 nm long pass dichroic mirror and a 590 nm long pass emission filter ensured proper illumination and detection wavelengths. An excitation intensity of 6.0 W/cm^2^ was used in all experiments. Finally, an Andor NEO sCMOS camera was used to record images. Single particle intensities were determined using ThunderSTORM^34^. For the single particle intensity measurements, the obtained data was directly analyzed in Mathematica. To obtain the average single particle intensities, for every sample, data obtained from at least 20 images was plotted in a histogram. The first peak in this histogram was attributed to the single particle intensity and a normal distribution was fitted to this peak to obtain the mean intensity and the standard deviation. For the bleaching measurements, a second analysis was performed in MatLab to trace the intensity of single particles from frame to frame. Furthermore, the data was filtered based on the single particle intensities determined from the first frame to remove clusters from the data set. The filtered data was averaged per frame to obtain bleaching curves.

### Cell culture

HeLa cells and HT1080 cells stably expressing LAMP-1-GFP were cultured in a 37 °C, 5% CO_2_ incubator, in T75 culture bottles (Corning). Cells were maintained in Dulbecco’s Modified Eagle’s Medium (DMEM; Gibco) supplemented with 10% fetal bovine serum, 2 mM L-glutamin, 100 U/mL penicillin, 100 *μ*g/mL streptomycin (referred to as complete DMEM). Cells were passaged when confluency reached 85% to 90%.

### 2D CLEM: Widefield and TEM imaging of fiducials on thin cryosections

HT1080 cells stably expressing LAMP-1-GFP were incubated in complete DMEM containing 5 nm diameter colloidal gold particles conjugated to bovine serum albumin (BSA-Au^5^) for 3 hours. Following incubation, cells were processed for cryosectioning according to previous protocol^35^. Briefly, cells were chemically fixed using formaldehyde and glutaraldehyde, scraped from the culture substrate and pelleted in 12% gelatin. Samples were infiltrated overnight in 2.3 M sucrose for cryoprotection, and plunge frozen in liquid nitrogen. 70 nm thick cryosections were sectioned and picked up on copper support grids coated with formvar and carbon. Sections were treated with DAPI (4 *μ*g/mL) diluted in PBS to label nuclei. After labelling, sections were washed with PBS, incubated with a diluted solution (1/500) of the fiducial markers in water, followed by rinses with PBS and dH_2_O. The grids were sandwiched between a microscope slide and a #1.5 coverslip in a drop of 50% glycerol in dH_2_O. Fluorescence imaging for DAPI, GFP and the fluorescent nanoparticles was performed with a Deltavision RT Core widefield microscope (GE Healthcare) equipped with a Cascade II EM-CCD camera (Photometrics), using a 100x/1.4 NA objective. Following fluorescent imaging, the sections were washed in dH2O, stained with uranyl acetate and embedded in methylcellulose as previously described^35^. ROIs determined in FM were retraced and imaged in a Tecnai T12 TEM (Thermo Scientific). Following imaging, the x and y positions of the fiducials in fluorescence data were registered using ThunderSTORM^34^. Fiducials not properly resolved in ThunderSTORM were not considered as reference points for registration of data. In TEM data, positions of the fiducials were registered manually using the center of the gold core. Correlation of fluorescence and TEM data based on the positions of the particles was performed using eC-CLEM^36^.

### 3D CLEM: Confocal and FIB-SEM imaging of endocytosed nanoparticles

Hela cells were grown on gridded glass coverslips, prepared as described by Fermie *et al*.^37^. Cells were incubated with fiducial markers at a concentration of 1 *μ*g/ml dissolved in complete DMEM and incubated for 3 hours, and fixed overnight in 1x PHEM buffer (60 mM PIPES, 25 mM HEPES, 10 mM EGTA, 2 mM MgCl_2_, pH = 6.9) containing 4% paraformaldehyde (Sigma) and 0.1% glutaraldehyde (Merck) at 4 °C. Following fixation, coverslips with cells were washed in 1x PHEM buffer and mounted in live-cell coverslip holders filled with 1x PHEM buffer to prevent dehydration of the samples. Fluorescence imaging was performed using a Zeiss LSM700 CLSM equipped with 63x/1.4 NA oil immersion objective. Nanoparticles were excited using the 555 nm laser line at 2% power. Z-stacks were collected with 200 nm step size. The position of cells relative to the grid of the coverslips was recorded using polarized light. Cells were prepared for electron microscopy according to a protocol described earlier^38^, with minor modifications. Briefly, samples were postfixed using 1% osmium tetroxide (w/v) with 1.5% potassium ferrocyanide (w/v) for 1 h on ice, incubated with 1% thiocarbohydrazide in dH_2_O (w/v) for 15 min, followed by 1% osmium tetroxide in dH_2_O for 30 min. Samples were en-bloc stained with 2% uranyl acetate in dH_2_O for 30 minutes and stained with Walton’s lead aspartate for 30 min at 60 °C. Dehydration was performed using a graded ethanol series. Samples were embedded in Epon resin and polymerized for 48–60 h at 65 °C. Polymerized resin blocks were removed from the glass coverslips using liquid nitrogen, mounted on aluminum stubs and rendered conductive using conductive carbon paint and a sputter coated layer of 5 nm Pt. Following sample preparation, automated serial imaging was performed using a Scios FIB-SEM (Thermo Scientific), according to a previously described workflow^37^. Briefly, trenches were prepared surrounding the region of interest using the FIB, after which automated serial imaging was performed using 5 nm isotropic voxels. Electron microscopy images were collected at an acceleration voltage of 2 kV and a current of 0.2 nA, using the T1 backscattered electron detector. Following imaging, correlation of fluorescence and FIB-SEM data was achieved by manual registration using Fiji and ec-CLEM^36^. FIB-SEM images are presented with inverted contrast, to resemble TEM contrast.

## Electronic supplementary material

Supplementary Information

## Data Availability

The datasets generated during and/or analysed during the current study are available from the corresponding author on reasonable request.
