# Supplementary material for: Fluorescently Labelled Silica Coated Gold Nanoparticles as Fiducial Markers for Correlative Light and Electron Microscopy
Source: Sci Rep. 2018 Sep 11;8:13625. doi: 10.1038/s41598-018-31836-1 (PMC6133918; doi:10.1038/s41598-018-31836-1)
Supplement: Supplementary file 1 — Supplementary Information [file 41598_2018_31836_MOESM1_ESM.pdf]

Supplementary Information

Fluorescently Labelled Silica Coated Gold Nanoparticles as Fiducial Markers for Correlative Light and Electron Microscopy

Jantina Fokkema[1], Job Fermie[1,2], Nalan Liv[2], Dave J. van den Heuvel[1], Tom O.M. Konings[1], Gerhard A. Blab[1], Andries Meijerink[3], Judith Klumperman[2] and Hans C. Gerritsen[1]

[1]Soft Condensed Matter and Biophysics, Debye Institute for Nanomaterials Science, Utrecht University, The Netherlands
[2]Section Cell Biology, Center for Molecular Medicine, University Medical Center Utrecht, Utrecht University, The Netherlands
[3]Condensed Matter and Interfaces, Debye Institute for Nanomaterials Science, Utrecht University, The Netherlands
[*]Correspondence and requests for materials should be addressed to H.C.Gerritsen@uu.nl

1 Synthesis of the fiducial markers

The particles were synthesized via the multistep procedure depicted schematically in figure S1. In the first step, citrate stabilized gold nanoparticles with a diameter of approximately 15 nm were synthesized in water via the extensively studied citrate reduction or Turkevich method [1, 2]. Next, the particles were coated with a nonionic polymer, polyvinylpyrrolidone or PVP, so that they could be transferred into ethanol following the procedure described by Graf *et al.* [3]. Once the particles were transferred to ethanol, they were coated with a fluorescently labelled silica shell via a seeded growth procedure similar to the procedure described by H. Giesche [4, 5]. This seeded growth procedure is based on the traditional Stöber method [6] to synthesize silica particles of a specific size obtained via the addition of silica precursor, TEOS, to mixtures of water, ammonia and alcohol.

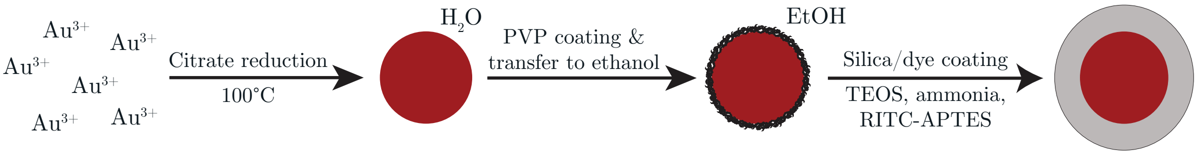

Figure S1: A schematic representation of the synthesis of the (rhodamine B labelled) silica coated gold nanoparticles.

Fluorescent dyes were covalently incorporated within the silica matrix during shell growth following the procedures described by A. Imhof *et al.* [7] for fluorescein isothiocyanate (FITC) and Verhaegh *et al.*[8] for rhodamine B isothiocyanate (RITC). Covalent incorporation is ensured by linking a fluorophore with an active amine reactive group such as an isothiocyanate group with an aminosilane; (3-aminopropyl)triethoxysilane or APTES. After coupling, this complex was introduced during the silica shell growth and part of it was incorporated within the silica shell.

1.1 Materials

Hydrogen tetrachloroaurate(III)trihydrate or chloroauric acid (ACS reagent) was obtained from ACROS Organics. Sodium citrate tribasic dihydrate, tetraethyl orthosilica or TEOS (reagent grade, 98%), polyvinylpyrrolidone or PVP (Average M_w 10.000 g mol^{-1}), ammonium hydroxide solution (ACS reagent, 28-30% NH_3 basis), rhodamine B isothiocyanate (mixed isomers) or RITC, 99% (3-Aminopropyl)triethoxysilane or APTES , dialysis tubing cellulose membrane (av. flat width 33 mm) and Vivaspin 20 centrifugal concentrator tubes (MWCO 100.000 Da) were purchased from Sigma-Aldrich. Absolute ethanol was purchased from Merck.

All glassware and stirring beans used for the gold synthesis were rinsed with aqua regia and mili-Q water prior to use. All remaining glassware was extensively cleaned with soap, water and ethanol prior to use. Milli-Q water was used in all the experiments.

1.2 Methods

Synthesis of 15 nm diameter gold cores
Gold and citrate stock solutions were prepared by transferring 0.1 g chloroauric acid and 0.1 g sodium citrate to two separate vials. 10 mL water was added to both vials to obtain 1% (w/v) stock solutions. In a typical synthesis, 300 mL water and 3 mL of the 1% (w/v) gold solution were transferred to a two necked round bottomed flask equipped with a condenser. This flask was placed in a 140°C oil bath to ensure homogeneous heating and was heated until boiling under constant vigorous stirring (600 rpm). When boiling commenced, 9 mL of the 1% (w/v) sodium citrate solution was added. Within the first few minutes after sodium citrate addition, the color of the solution gradually changed from blue to purple to deep red indicating particle formation. After 15 minutes boiling no change in color was observed anymore and the deep red solution was cooled down to room temperature.

PVP functionalization and transfer of the particles to ethanol
120 μL of a 10% (w/v) PVP solution (1 g in 10 mL water) was added per 5 mL of gold nanoparticle solution under constant stirring. The obtained solution was stirred for at least 12 hours to ensure functionalization of the gold nanoparticles. After 12 hours, the solution was centrifuged 15 minutes at 15.000 rcf in 5 mL eppendorf tubes.

The supernatant (water) was removed as much as possible with a glass pipette and the particles were redispered in an equal amount of ethanol. Homogenization of the sample was ensured by placing the obtained solution in a sonication bath ($\sim$ 1 minute).

APTES-dye coupling

To perform the APTES-dye coupling reaction, 10 mg RITC and 1 mL absolute ethanol were transferred to a vial. This solution was stirred and 8.3 μL APTES was added. The vial was wrapped in aluminum foil to protect the fluorophore from photobleaching and stirred for 16 hours. The APTES-dye ratio during this coupling reaction was calculated such that there was a two times excess of APTES compared to the amount of fluorophore. Different dilutions of this dye solution in absolute ethanol were used to synthesize particles labelled with different dye labelling densities. Solutions with relative dye concentrations ranging from 0 to 30 (labelled as [Dye] = 0 - 30) were prepared by preparing dilutions of 0 to 30 vol% of APTES-dye solution in ethanol. For example, to obtain a relative dye concentration of 30, 300 μL APTES-dye solution (30 vol%) and 700 μL absolute ethanol (70 vol%) were transferred to a vial.

Coating of the gold nanoparticles with a fluorescently labelled silica shell

15 mL solution of PVP stabilized gold nanoparticles was transferred to a 20 mL closed vial. Under continuous stirring (700 rpm), 1500 μL 28-30% ammonium hydroxide solution was added to this solution. Next, 30 μL of a 10 vol% of TEOS solution in absolute ethanol was added to grow a first, very thin, silica layer. This layer helps to stabilize the particles since the immediate addition of APTES-dye solution results in the formation of clusters of gold nanoparticles. Furthermore, this layer serves as a spacer layer between the gold cores and the fluorophores embedded in the silica shell. 60 minutes after the first TEOS addition an additional 150 μL of the 10 vol% TEOS solution was added. After approximately one minute of stirring, 150 μL of diluted APTES-dye solution was added as well. Both additions were repeated with 300 μL after 90 minutes of stirring. Finally, after another 90 minutes, 60 μL of 10 vol% TEOS was added to grow a final stabilization layer around the fluorescently labelled particles.

60 minutes after the final addition, the reaction mixture was transferred to a dialysis bag inside a 40 mL vial filled with ethanol. The solution was gently stirred and the ethanol was replaced three times in a time span of 36 hours. After 36 hours, further cleaning of the particles was performed via repeated centrifugation in 100.000 MWCO vivaspin tubes.

2 A calibration line to synthesize particles of a desired size

To synthesize particles of a desired size, a multistep silica coating of the gold nanoparticles was performed. Increasing volumes of TEOS were added to 5 mL of PVP functionalised gold nanoparticles in a solution of water, ammonia and absolute ethanol. 90 minutes after every addition, TEM samples were prepared. In figure S2 representative TEM images of these samples are shown.

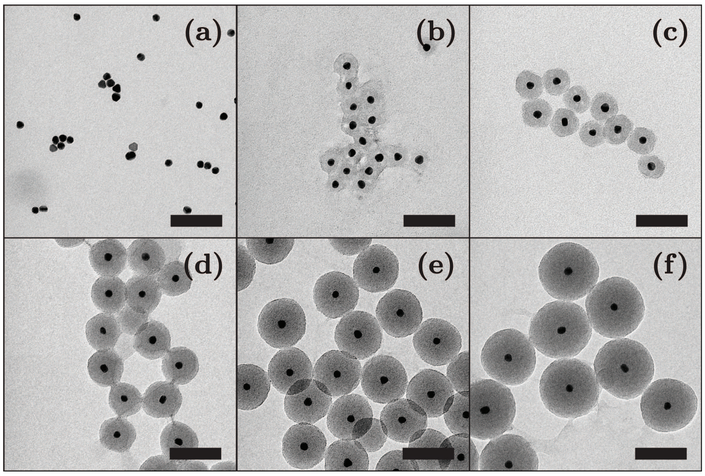

Figure S2: Representative TEM images of samples taken at different stages of silica coating. Sample (a) is prepared before TEOS addition. From (b) to (f) volumes of 20, 40, 80, 160 and 320 μL of 10 vol% TEOS are added.

From the TEM images it becomes clear that the silica shell is already homogeneous after the first TEOS addition (b). Furthermore, one can conclude that the thickness of the silica shell is increasing from (b) to (f) after every TEOS addition. Average particle diameters of all samples were determined by measuring the diameter of 100 particles, corresponding size histograms are presented in figure S3a. In figure S3b the average particle diameters are plotted versus the cube root of the total volume of added 10 vol% TEOS. From this plot it becomes evident that there is a linear relationship between these two parameters after the first TEOS addition. This is also in line with expectations since TEOS is converted directly into silica. Therefore, if silica grows only on the existing particles, there should be a linear relationship between the volume of added TEOS and the increase in volume of the particles. This linear relationship demonstrates that particles with every desired size can be synthesized with high control simply by varying the amount of added TEOS. From figure S3b it can be derived how much TEOS should be added to synthesize particles of a desired size starting from the same solution of gold particles.

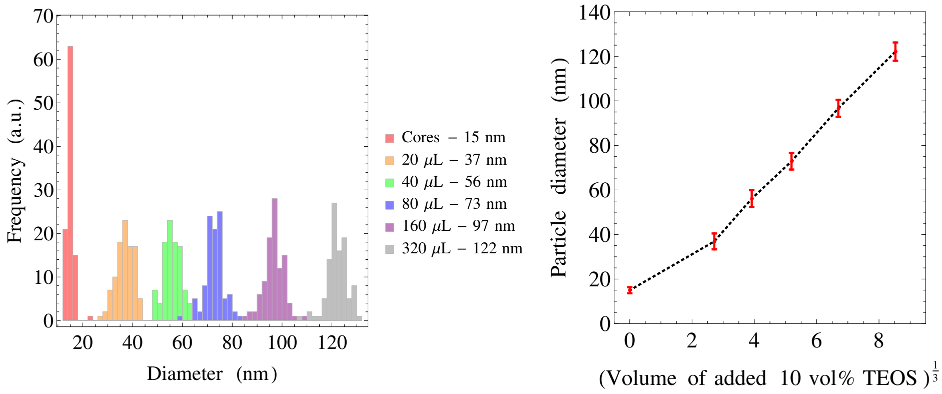

(a)

(b)

Figure S3: Size histograms (a) and a plot (b) showing the linear relationship between the cube root of the added volume of TEOS and the particle diameter.

3 2D-CLEM experiment: Recognition of the fiducials

In this section it is demonstrated how the unique signature of the particles can be used to identify the fiducials in EM. To do so, specific areas of the EM image of the 2D-CLEM example presented in the main text are enlarged in figure S4. This demonstrates that already in this low magnification image the core-shell structure can be used to distinguish between fiducials (encircled in green) and other dark spot that are excluded as fiducials (encircled in orange). This is also confirmed by FM data since no fluorescence is observed from the dark spots that are excluded as fiducials.

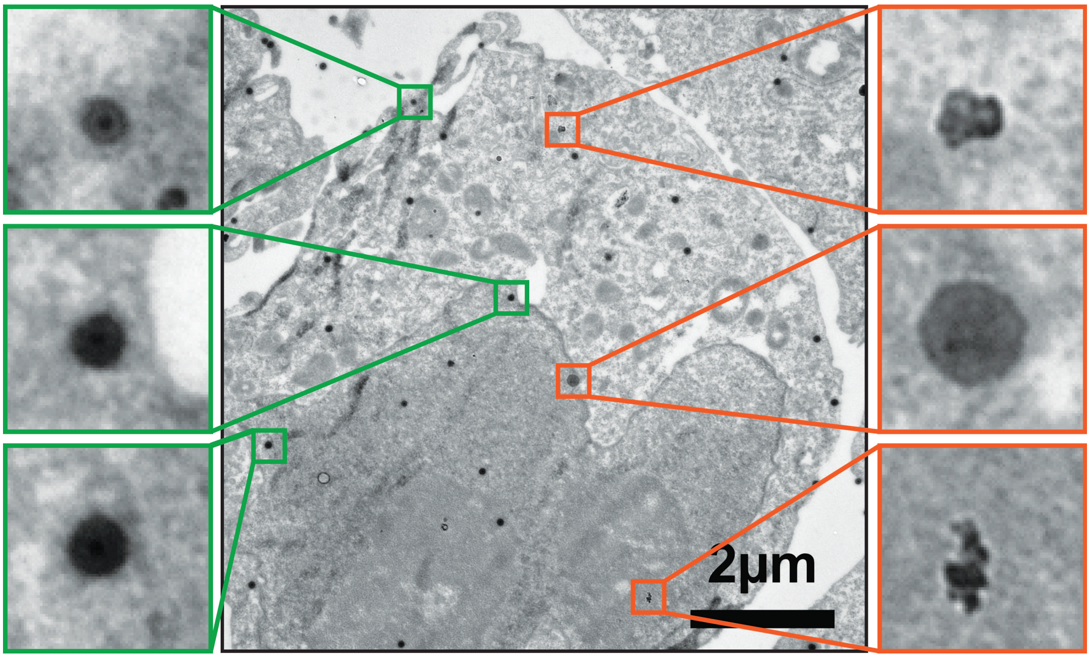

Figure S4: Low Magnifcation TEM image of 81.9 nm diameter fiducials on top of a 70 nm thick cryosection of HT1080 cells stably expressing LAMP-1-GFP.

This becomes even more apparent in the higher magnification EM image of the same region of interest included in figure S5. Again, the particles encircled in green correspond to fiducials whereas regions encircled in orange are excluded as fiducials because of the absence of the distinct core-shell structure.

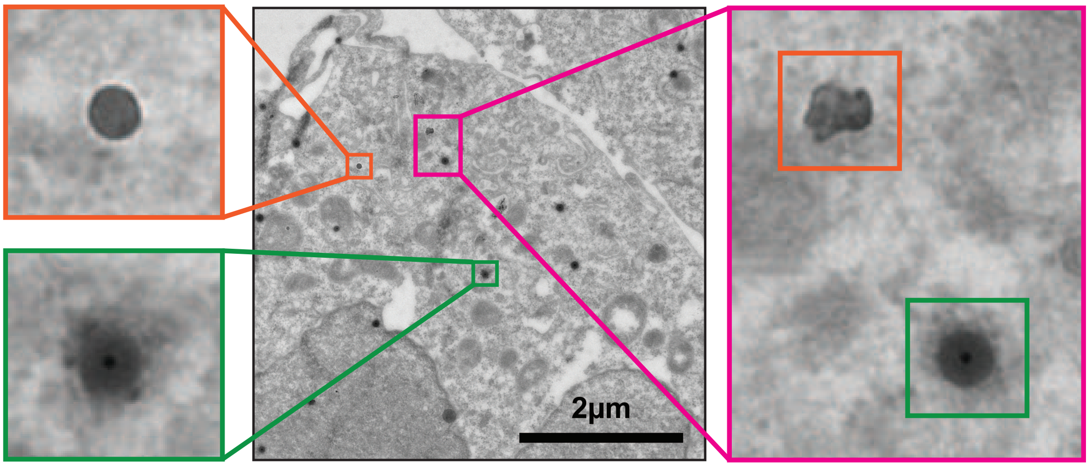

Figure S5: TEM image of 81.9 nm diameter fiducials on top of a 70 nm thick cryosection of HT1080 cells stably expressing LAMP-1-GFP.

References

[1] J. Turkevich, P. C. Stevenson, and J. Hillier, "A study of the Nucleation and Growth Processes in the Synthesis of Colloidal Gold," *Discuss. Faraday Soc.*, vol. 11, no. c, pp. 55–75, 1951.

[2] S. D. Perrault and W. C. W. Chan, "Synthesis and Surface Modification of Highly Monodispersed, Spherical Gold Nanoparticles of 50-200 nm.," *Journal of the American Chemical Society*, vol. 131, pp. 17042–3, dec 2009.

[3] C. Graf, D. L. J. Vossen, A. Imhof, and A. van Blaaderen, "A General Method To Coat Colloidal Particles with Silica," *Langmuir*, vol. 19, pp. 6693–6700, aug 2003.

[4] H. Giesche, "Synthesis of Monodispersed Silica Powders I. Particle Properties and Reaction Kinetics," *Journal of the European Ceramic Society*, vol. 14, pp. 189–204, jan 1994.

[5] H. Giesche, "Synthesis of Monodispersed Silica Powders II. Controlled Growth Reaction and Continuous Production Process," *Journal of the European Ceramic Society*, vol. 14, pp. 205–214, jan 1994.

[6] W. Stöber, A. Fink, and E. Bohn, "Controlled Growth of Monodisperse Silica Spheres in the Micron Size Range," *Journal of colloid and interface science*, vol. 69, pp. 62–69, 1968.

[7] A. Imhof, M. Megens, J. J. Engelberts, D. T. N. de Lang, R. Sprik, and W. L. Vos, "Spectroscopy of Fluorescein (FITC) Dyed Colloidal Silica Spheres," *The Journal of Physical Chemistry B*, vol. 103, pp. 1408–1415, mar 1999.

[8] N. A. M. Verhaegh and A. V. Blaaderen, "Dispersions of Rhodamine-Labeled Silica Spheres: Synthesis, Characterization, and Fluorescence Confocal Scanning Laser Microscopy," vol. 96, no. 9, pp. 1427–1438, 1994.
